# Association between sleep disturbance and mental health of healthcare workers: A systematic review and meta-analysis

**DOI:** 10.3389/fpsyt.2022.919176

**Published:** 2022-07-29

**Authors:** Ying Liu, Qin Zhang, Fugui Jiang, Hua Zhong, Lei Huang, Yang Zhang, Hong Chen

**Affiliations:** ^1^West China School of Nursing/West China Hospital, Sichuan University, Chengdu, China; ^2^Department of Postgraduate Students, West China School of Medicine/West China Hospital, Sichuan University, Chengdu, China; ^3^Sichuan Provincial Center for Mental Health, Sichuan Academy of Medical Sciences & Sichuan Provincial People’s Hospital, Chengdu, China; ^4^Department of Environmental Health and Occupational Medicine, West China School of Public Health and West China Fourth Hospital, Sichuan University, Chengdu, China; ^5^Department of Occupational Hazard Assessment, West China School of Public Health and West China Fourth Hospital, Sichuan University, Chengdu, China; ^6^Department of Periodical Press and National Clinical Research Center for Geriatrics, West China Hospital, Sichuan University, Chengdu, China; ^7^Chinese Evidence-Based Medicine Center, West China Hospital, Sichuan University, Chengdu, China

**Keywords:** healthcare workers, mental health, sleep, COVID-19, systematic review

## Abstract

**Objectives:**

Sleep disturbance and mental health are challenges for healthcare workers (HCWs). Especially during the COVID-19 pandemic, they experienced more severe sleep and mental health problems. However, the association between sleep disturbance and the mental health of HCWs is still controversial. This study aimed to systematically review the relationship by conducting a systematic review and meta-analysis.

**Method:**

Two researchers retrieved the literature from Web of Science, PubMed, EMBASE, CINAHL, Psyclnfo, and Cochrane Library from the establishment of the databases until November 20, 2021. We used the New Castle-Ottawa Scale (NOS) and Agency for Healthcare Research and Quality (AHRQ) to evaluate the risk of bias in prospective research and cross-sectional research, respectively. The major exposure was HCWs’ sleep disturbance, and the major outcome was mental health. The correlation coefficients (*r*), regression coefficients (β) and odds ratios (OR) of the included studies were integrated.

**Result:**

Fifty-nine studies were included for qualitative analysis, of which 30 studies could be combined and entered into quantitative analysis. There were 23 studies during the COVID-19 pandemic among the 59 included studies. The results of the meta-analysis showed that the correlation coefficient between sleep disturbance and mental health was 0.43 (95% CI: 0.39–0.47). HCWs with sleep disturbance had a 3.74 (95% CI: 2.76–5.07) times higher risk of mental health problems than those without sleep disturbance. The correlation coefficient during the COVID-19 epidemic was 0.45 (95% CI: 0.37–0.53), while it was 0.40 (95% CI: 0.36–0.44) during the non-epidemic period. Subgroup analysis compared the OR results in epidemic and non-epidemic periods of COVID-19, which were 4.48 (95% CI: 2.75–5.07) and 3.74 (95% CI: 2.74–7.32), respectively.

**Conclusion:**

Sleep disturbance and mental health problems were positively correlated among HCWs. Particularly in the COVID-19 pandemic, more attention should be given to this issue.

## Introduction

Healthcare workers (HCWs) who encourage and improve most of the population’s lives are the backbone of each health system (1). Approximately 43,500,000 registered HCWs worldwide were reported from World Health Organization (WHO) publication data (2). Meanwhile, they play a key role in the control of every infectious and fatal disease. The screening, identification and treatment of clients are the responsibilities of HCWs, who are providing services for disease control 24 h a day (3). Therefore, sleep disturbances are health challenges in HCWs resulting from rotating or extended-duration shifts and other complicated work situations (4). The prevalence of these symptoms among clinical nurses was 63.9% in a cross-sectional study in China (5), while in Iran it was found that 53.1% of HCWs had sleep disturbances (6). COVID-19, which is an aggressive event in the global health system, causes more serious sleep problems in HCWs. A survey of 987 people stated that 58.8% of physicians and nurses suffered from sleep disturbance (7). In addition, 26.67% of front-line HCWs experienced severe insomnia (8).

However, HCWs, who are exposed to disease, face the transmission of infection to family members, a shortage of personal protective equipment (PPE), extended working hours and decisions about allocating limited resources to patients, have a risk of adverse mental health (9–11). It appealed that depression and anxiety prevalence among HCWs were 22.8 and 23.2%, respectively, derived from a meta-analysis including 33062 subjects (12). Meanwhile, existing literature has shown that HCWs represent the highest risk groups of depressive symptoms, suicide, and post-traumatic stress disorder (PTSD) among the general population (13–15). It leads to a high incidence of low job satisfaction, absenteeism and turnover directly (13). Hence, we should pay attention to HCWs’ mental health problems. There are numerous factors affecting mental health. Some research has shown that mental health situations can be attributed to individual, demographic, environmental, shift work, chemical and biological hazards, deficiencies in resiliency, coping strategies and sleep (16–18).

The correlation between sleep and mental health has been discovered in recent years (19, 20). It was reported that pregnant women with sleep disturbance in the 3rd trimester have a 5.27 times higher risk of depression (20). Nurses with sleep disorders had a 1.2 (95% confidence interval, CI: 1.13–1.27) times higher risk of anxiety than those without sleep problems (21). Furthermore, Yost et al. found that sleep disturbance level was positively correlated with burnout level among HCWs (*r* = 0.42) (22). Meanwhile, researchers have also explored the mechanism of how sleep impacts individual mental health (19). Nevertheless, there is doubt about the association between sleep disturbance and the mental health of HCWs. There is also a lack of systematic review. Therefore, we performed this research to provide evidence of enhancing HCWs’ mental health.

## Materials and methods

This systematic review was conducted and reported in line with Preferred Reporting Items for Systematic Reviews and Meta-Analyses (PRISMA) recommendations (23). The study was registered with the International Prospective Register of Systematic Reviews (PROSPERO registration number CRD42021286163).

### Search strategy

First, two researchers retrieved the literature from Web of Science, PubMed, EMBASE, CINAHL, Psyclnfo, and Cochrane Library from the establishment of the database until August 31, 2021. We updated the database search to November 15, 2021. The search strategy combined Medical Subject Headings (MeSH) and free text terms to identify the relevant articles. Keywords for the search were “sleep” OR “sleep wake disorder” OR “insomnia” OR “nightmare” OR “parasomnia” OR “circadian” OR “dyssomnia*” OR “chronotype” AND “healthcare workers” OR “Health Personnel” OR “Medical staff” OR “Health Care Provide” OR “Nurse” OR “Physician” AND (“Mental health” OR “mental disorders” OR “anxiety” OR “depression” OR “Stress, Psychological”). The detailed process of each database is supported in Supplementary material.

### Inclusion criteria

Studies were included by applying the following population-exposure-control-outcome-study design (PECOS):

(a) Population—studies of HCWs, including doctors, physicians, residents, nurses, nursing assistants, pharmacists, laboratory technologists, physical therapists, technicians, assistant healthcare staff, and other HCWs, which were not specific occupations but were classified as HCWs in the included studies.

(b) Exposure and control—data on HCWs with and without sleep disturbance during the study period. Sleep disturbance was defined as low sleep quality or short sleep duration. Sleep qualities were evaluated by subjective self-rating scales, such as the Pittsburgh Sleep Quality Index (PSQI), the Insomnia Severity Index (ISI), The PROMIS Sleep Disturbance Short Form, The Bergen Insomnia Scale (BIS), Symptom Checklist 90 (SCL-90) sleep part, Sleep Quality Scale (0–10 score), Sleep Habits Questionnaire (CHAS), Sleep Disorder Screening Questionnaire, Sleep Condition Indicator (SCI), “How many nightmares did you have that woke you up?,” “Health status (grade): “Do you have difficulty in sleeping (no/slight/serious),” Hamilton Depression Rating Scale (SIGH-D) sleep part, and some items designed by researchers. For example, what do you think of the quality of sleep? The answer was from good to very bad.

(c) Outcome—studies on mental health measurement of HCWs. Types of mental health included anxiety, burnout, depression, distress, mental health complaints, post traumatic symptom disorder (PTSD) and stress. Research tools included the Depression, Anxiety, and Stress Scale (DASS-21), Self-Rating Anxiety Scale (SAS), Short Health Anxiety Inventory (SHAI), General Anxiety Disorder-7 (GAD-7), Hospital Anxiety and Depression Scale (HADS), Goldberg Anxiety and Depression Scale (GADS), Goldberg’s General Health Questionnaire (GHQ-28), and Beck Anxiety Inventory (BAI) were used to measure anxiety levels. Burnout was estimated by the Maslach Burnout Inventory-Human Services Survey (MBI-HSS), Professional Quality of Life Scale (ProQOL-CN), Maslach Burnout Inventory-Short Form (MBI-SF), Link Burnout Questionnaire (LBQ), Index Chronic Fatigue Scale (SSICFS), 11-item Chalder Fatigue Scale (CFS), Maslach Burnout Inventory-General Survey (MBI-GS), Copenhagen Burnout Inventory (CBI), and Swedish Occupational Fatigue Inventory (SOFI). The Center for Epidemiologic Studies Depression Scale (CES-D), DASS-21, Patient Health Questionnaire-9 (PHQ-9), Core Symptoms of Depression, HADS, Goldberg’s General Health Questionnaire (GHQ-28), GADS, and Self-Rating Depression Scale (SDS) were used to assess depression levels. General Health Questionnaire-12 (GHQ-12) was used to measure distress. The MHI-5 screening test was applied to evaluate mental health complaints. The Impact of Event Scale (IES-R) and Post-traumatic Stress Disorder Checklist for DSM-5 (PCL-5) assessed PTSD. Stress level was measured by DASS-21, IES-R, Perceived Stress Scale (PSS), Diary of Ambulatory Behavioral States, and other items designed by researchers. That was “‘I felt stressed’, rated on a scale of 0 (not at all) to 4 (extremely)” and “instrument containing 10 Likert scale questions by investigator.”

(d) Study design—observational research including cohort study, case-control study and cross-sectional studies.

### Exclusion criteria

The exclusion criteria were as follows: (a) Studies were not reported in English; (b) Reviews, letters, case reports, protocols, conference abstracts and any article without full text; (c) Studies’ results without data; (d) Only univariate analysis for statistics; and (e) Demonstration of the effects of mental health on sleep disturbance.

### Data extraction

First, two researchers independently read the titles and abstracts when screening relevant studies. Subsequently, they read the full texts of the literature proved potentially eligible after title and abstract screening. In the process of reading, research on the inability to analyse the correlation between sleep and mental health was excluded. To collect data from each included study, the main examiner created a unified information extraction table based on the research questions. The following data were extracted from each study: (a) Study characteristics: publication year, research time, first author name, country, study design, multi-center study or single-center study, total population included, included population sample, mean age, sex ratio, and occupation; (b) Other data: COVID-19 pandemic or not, sleep disturbance type, diagnostic sleep scale, the cut-off of the scale (if it had), the number of sleep measurements, the case number, mental health problem type, the number of mental health measurements, the case number (for mental health measurements), diagnostic mental health scale and the cut-off of the scale (if it had); and (c) Statistical data: *r*, 95% CI of *r*, OR, 95% CI of OR, β, and 95% CI of β. The data were extracted directly into the table and cut or paste functions were used as much as possible to reduce the possibility of transcription errors. To ensure completeness, each study was reviewed at least twice by the principal reviewer.

### Risk of bias assessment

Two researchers independently evaluated the quality of the included studies according to the 8 items of the Newcastle-Ottawa Scale (NOS) for cohort studies and case-control studies. The NOS scale is composed of three dimensions and eight items with a full score of 9 points. A score less than 4 was defined as a low-quality study. A score of 4–6 was defined as a medium-quality study, and a score of ≥ 7 was defined as a high-quality study. The higher the score, the lower the risk of bias (22, 23). Quality evaluation of the cross-sectional study adopted the scale recommended by the Agency for Healthcare Research and Quality (AHRQ) (24). The scale covers 11 items. The answers include yes, no, and unclear. If the answer was “yes,” the item was judged as a 1 point. A higher score means a higher quality level. Different scores correspond to different quality levels: low quality = 0–3; medium quality = 4–7; and high quality = 8–11. If the two researchers disagreed with the literature inclusion and quality evaluation, the third researcher arbitrated. The details about the risk of bias evaluation are shown in Supplementary material.

### Statistical analysis

Statistical analysis was performed by using Stata 15.0. The correlation coefficient (*r*), regression coefficient (β) and odds ratio (OR) were calculated. Since *r* does not obey the normal distribution (25), we converted it to Fisher’s *Z* (*Zr*) for the integration of statistics. To facilitate the combination of statistics, the regression coefficient (β), which came from the logistic regression model, was converted into OR. The rule for conversion was that performed according to the β power of e equal to OR. Then, the ORs were combined. If the evaluation result adopted reverse scoring, the result score was converted (26), and the data were merged. Only descriptive analysis was performed for studies in which effect sizes could not be combined.

Heterogeneity among the trials was assessed using the Chi-square test (*P* < 0.10, defined as indicating significant heterogeneity) or *I*^2^ (> 50% heterogeneity was indicated). If substantial heterogeneity existed, we analysed the sources of heterogeneity and decided if a random effect model was applied. Otherwise, a fixed effect model was applied. Subgroup analysis was performed to determine whether different kinds of sleep disturbance and mental health were affected during COVID-19. The studies whose research time was the pandemic period were included in the COVID-19 pandemic subgroup. Sensitivity analysis was conducted to evaluate the robustness of the results by excluding individual studies for forest plots. Publication bias was evaluated by asymmetry of the funnel plot.

## Results

### Study selection and characteristics

**FIGURE 1:**
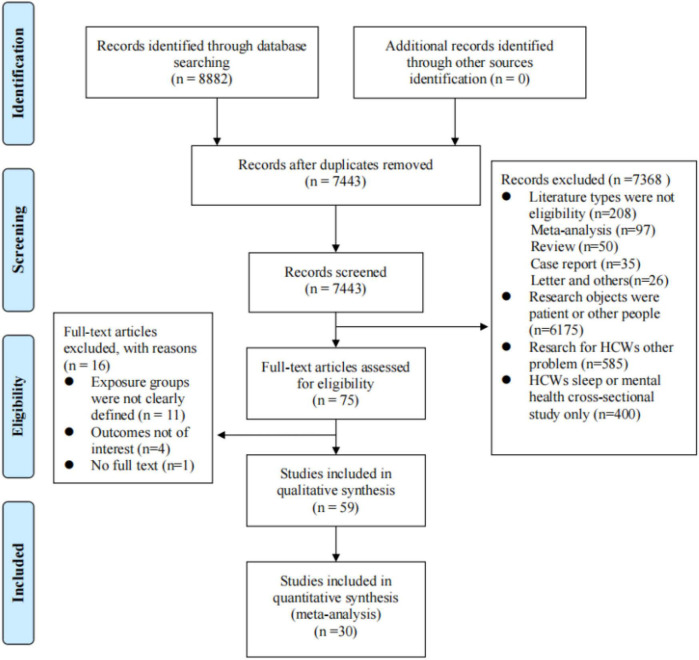
PRISMA flow chart illustrating the selection process of literature.

The literature retrieval process based on the PRISMA flow diagram is shown in Figure 1. A total of 8,882 records were retrieved initially. After removing duplication, 74,443 records remained. A total of 7368 records were excluded after reading the abstract, and 75 studies were selected to be included in the full text screening. Finally, 59 studies were included for analysis, of which 30 studies could be combined and entered into quantitative analysis. The reasons why 29 studies could not be qualitatively analysed were as follows: (a) The statistical model of the studies using β as the research result did not use logistic regression, so β could not be transformed and merged (*n* = 19). Research on correlation analysis using structural equation models was also excluded (*n* = 4); (b) Sleep included in logistic regression was defined as a continuous variable (*n* = 4); and (c) There was no 95% confidence interval for β or OR (*n* = 2).

The basic characteristics of the studies are shown in Table 1. We found that research came from 20 countries: America (10), Brazil (1), Canada (1), China (20), Egypt (4), Ethiopia (1), Iran (2), Italy (2), Japan (2), Norway (1), Peru (1), Philippines (1), Poland (1), Romania (1), Saudi Arabia (2), Singapore (1), Spain (2), Sweden (1) andTurkey (5). The research sites were concentrated in general hospitals, and the research subjects were mainly physicians and nurses. A total of 41,566 samples were included, of which 71.35% were females (*n* = 29,659). There were 23 studies conducted during the COVID-19 pandemic.

**TABLE 1:** Characteristics of all studies included in this study (*n* = 59).

References	Study location	Sample size^a^	Sex ratio (male/female)	Sample age (years)^b^ in Survey Year	Sample characteristics (follow-up status for longitudinal studies)	Result of risk bias evaluation^c^
**Prospective study**						
Hillhouse et al. (27)	America	46	31/15	30.6 ± 4.7	Residents (first residency year at beginning, and followed up with four repeated measures for one year and a half.)	7
Sørengaard et al. (21)	Norway	1688	174/1815	38.0 ± 8.3	Nurses (followed up with three repeated measure from 2012 to 2014)	8
Wang et al. (28)	China	1531	12/1496 (12 missing)	/	Nurses (not mentioned)	6
Fang et al. (29)	America	1269	919/1196	27.46 ± 2.43	Interns (Conducted the first survey before the internship initiated. In addition, followed up in the average of 17 (± 12) and 115 (± 111) days for tow repeated measures.)	8
**Cross-sectional studies**						
Ruggiero et al. (30)	America	128	0/402	44.9 ± 8.3	Female nurses	7
Lu et al. (33)	Philippines	135	20/105	32.28	Nurses	3
Peterson et al. (32)	Sweden	3719	/	47.0 ± 9.0	HCWs	8
Rutledge et al. (33)	America	185	/	27.9 ± 2.0/29.4 ± 2.0/35.9 ± 5.6	Physician	6
Stucky et al. (34)	America	304	98/206	30.2 ± 4.5/39.3 ± 9.8	Physician and Nurses	6
Sun et al. (35)	China	1134	539/595	46.8 ± 11.09/46.7 ± 10.85	Doctors	6
Aldrees et al. (36)	Saudi Arabia	348	250/98	35 ± 9.8	Resident physician	7
Yost et al. (22)	America	48	32/16	30.90 ± 3.40	Osteopathic otolaryngology residents	6
Chin et al. (37)	China	1084	0/1084	31.9 ± 8.0	Nurses	6
Qiao et al. (38)	China	492	147/345	33.77 ± 8.63	HIV/AIDS healthcare workers	7
Koyama et al. (39)	Japan	4737	1098/3639	/	HCWs	8
Vilchez-Cornejo et al. (40)	Peru	402	212/190	/	Medical Internships	6
Cai et al. (41)	China	1608	0/1608	32.25 ± 8.55	Female medical personnel	8
Vidotti et al. (42)	Brazil	502	48/454	/	Nurses	8
Wang et al. (43)	China	1044	94/950	/	Nurses	7
Ibrahim et al. (44)	Saudi Arabia	977	6/971	32.0 ± 7.0	Nurses	8
Dai et al. (45)	China	865	17/848	32.49 ± 10.35/28.33 ± 5.76	Nurses	9
Ghasemi et al. (46)	Iran	162	28/134	32.1 ± 7.5	Nurses	5
Aydin Sayilan et al. (47)	Turkey	267	66/201	28.03 ± 5.99	Nurses (during COVID-19 pandemic)	9
Youssef et al. (48)	Egypt	540	294/246	37.30 ± 9.20	HCWs (during COVID-19 pandemic)	4
Yin et al. (49)	China	371	143/228	35.30 ± 9.48	HCWs (during COVID-19 pandemic)	5
Weaver et al. (4)	America	1141	/	/	HCWs	5
Ji et al. (50)	China	380	79/301	28.1 ± 3.9	Pediatrics residents	8
Furihata et al. (51)	Japan	2482	0/2482	31.2 ± 8.9	Nurses	9
Cheng et al. (52)	China	534	94/440	/	HCWs (during COVID-19 pandemic)	5
Higgins et al. (53)	America	274	24/250	26.0-35.0	Nurses	9
Ng et al. (54)	China	447	252/195	34.1 ± 6.0	Doctors	8
Ding et al. (55)	China	1068	0/1068	32.0 ± 8.0	Female nurses	7
Eva et al. (56)	Spain	511	114/397	40.92 ± 9.23	HCWs	4
Tu et al. (57)	China	100	0/100	34.44 ± 5.85	HCWs (during COVID-19 pandemic)	6
Magnavita et al. (58)	Italy	592	175/417	/	HCWs (during COVID-19 pandemic)	9
**Cross-sectional studies**						
Korkmaz et al. (59)	Turkey	140	61/79	35.6 ± 8.7/30.7 ± 6.2	HCWs (during COVID-19 pandemic)	4
Secosan et al. (60)	Romania	126	45/81	/	HCWs (during COVID-19 pandemic)	8
Teo et al. (61)	Singapore	122	32/90	34 (21,73)	HCWs (during COVID-19 pandemic)	9
Tasdemir et al. (62)	Turkey	435	191/244	36.76 ± 7.58	HCWs (during COVID-19 pandemic)	6
Wang et al. (63)	China	562	118/444	35.00 (34.00, 36.00)	Nurses (during COVID-19 pandemic)	9
Mousavi et al. (64)	Iran	321	84/236	33.5 ± 7.65	HCWs (during COVID-19 pandemic)	5
Simonetti et al. (65)	Italy	1005	342/663	40.2 ± 10.80	Nurses (during COVID-19 pandemic)	7
Zhang et al. (13)	America	1060	372/689	47.28 ± 11.96	HCWs	6
Zhang et al. (66)	China	401	124/277	/	HCWs (during COVID-19 pandemic)	8
Yitayih et al. (67)	Ethiopia	249	118/131	27.40 ± 4.10	HCWs (during COVID-19 pandemic)	6
Chen et al. (68)	China	597	72/525	35	HCWs (during COVID-19 pandemic)	5
Kandemir et al. (69)	Turkey	194	56/138	29.99 ± 7.12	Nurses (during COVID-19 pandemic)	5
Aydin et al. (70)	Turkey	1011	332/679	35.67 ± 8.61	HCWs	8
Chang et al. (71)	China	1230	391/839	26.07 (25.88, 26.25)/25.99 (25.79, 26.20)	Resident physician	10
Mokros et al. (72)	Poland	54	16/38	34.81 ± 11.41/39.39 ± 12.10	Physical therapists	3
Abdelghani et al. (73)	Egypt	218	62/156	39.5 ± 8.5	HCWs (during COVID-19 pandemic)	6
Abu-Elenin (74)	Egypt	237	138/99	38.2 ± 6.2	Physicians (during COVID-19 pandemic)	6
Pang et al. (18)	China‘	282	32/250	31.61 ± 7.60	Nurses (during COVID-19 pandemic)	7
Abbas et al. (75)	Egypt	381	162/219	29.47 ± 5.49	ICU HCWs	8
Olagunju et al. (76)	Canada	303	183/120	38.8 ± 8.9	HCWs (during COVID-19 pandemic)	4
Hsieh et al. (77)	China	248	0/248	32.98 ± 8.25	Psychiatric Nurses	8
Jiang et al. (78)	China	569	228/341	34.0 ± 8.0	HCWs (during COVID-19 pandemic)	8
Geng et al. (79)	China	317	96/221	/	HCWs (during COVID-19 pandemic)	8
Garcia et al. (80)	America	389	32/360	39.54 ± 11.15	Nurses	6

^a^The final sample size in the cohort; ^b^Age: −x or−x ± s or Median (interquartile range, IQR); ^c^The Newcastle–Ottawa Scale (NOS) evaluated cohort-study. Agency for Healthcare Research and Quality (AHRQ) evaluated the cross-sectional study; /: Not reported; HCWs: Healthcare workers.

### Risk of bias assessment

The overall quality evaluation results of the study are shown in the last column of Table 1. A total of 59 observational studies were included in this systematic review, of which 55 were cross-sectional studies and 4 were cohort studies. In the cohort study, there were 3 studies (75.00%) with a quality evaluation score > 7, suggesting that most studies were of high quality. Among the 55 cross-sectional studies, 2 (3.65%) were low-quality studies, 31 (56.35%) were moderate-quality studies, and only 22 (40.00%) were high-quality studies.

### Association between sleep disturbance and mental health

Measures of sleep disturbance and mental health in the included studies are shown in Table 2. The most commonly used tools for measuring sleep disorders were the PSQI and ISI. The main tools for measuring mental health were the MBI, PHQ-9 and HAD. The statistical methods used focused on Pearson correlation, multiple linear regression and logistic regression.

**TABLE 2:** Measures of sleep disturbance and mental health in the included studies (*n* = 59).

References	Measures of sleep disturbance	Adjustment factors in the model	Measures of mental health	Statistical model
**Prospective study**				
Hillhouse et al. (27)	Sleep hours	Gender, specialty and citizenship (US versus non-US)	Perceived Stress Scale (PSS)	Hierarchical regression
Sørengaard et al. (21)	The Bergen Insomnia Scale (BIS)>2	/	Hospital Anxiety and Depression Scale (HADS)	Structural equation modelling
Wang et al. (28)	Pittsburgh Sleep Quality Index(PSQI) >7	Age, marital status, offspring status, current work tenure, professional status and depression status.	The Patient Health Questionnaire-9 (PHQ-9) (item No.9)>1	Multiple logistic regression models
Fang et al. (29)	24 h total sleep time (TST)	Age, gender, bedtime median and Wake time median	PHQ-9	Multivariable linear regression models
**Cross-sectional studies**				
Ruggiero et al. (30)	PSQI	/	Standard Shiftwork Index Chronic Fatigue Scale (SSICFS)	Simultaneous Multiple Regression
Lu et al. (31)	Designed by researchers: choose sleep disturbance or not	/	Maslach Burnout Inventory Human Services Survey (MBI-HSS)	Multiple linear regression
Peterson et al. (32)	Designed by researchers:three items	/	The Hospital Anxiety and Depression Scale (HAD)	Pearson correlation
Rutledge et al. (33)	PSQI	/	Diary of Ambulatory Behavioral States	Multiple linear regression
Stucky et al. (34)	Sleep quality scale>0 and sleep hours per day	Age, gender, familiarity with patients, average patientload and number of admissions in 24 hours	Study-developed instrument containing 10 Likert scale questions	Multiple linear regression
Sun et al. (35)	Health status (grade): Do you have difficulty in sleeping (no/slight/serious)	Age	Zung Self-Rating Anxiety Scale (SAS)	General linear regression
Aldrees et al. (36)	Sleep hours <6 h per day	/	Maslach Burnout Inventory (MBI)	Multiple logistic regression analysis
Yost et al. (22)	Sleep hours per day	/	MBI-HSS	Spearman correlation
Chin et al. (37)	Sleep hours <6 h per day	/	Modified Chinese version of the Copenhagen Burnout Inventory (C-CBI)	Multiple logistical regression
Qiao et al. (38)	Symptom Checklist 90 (SCL-90): sleep part>2	/	Maslach Burnout Inventory-General Survey (MBI-GS)	Multiple logistic regression
Koyama et al. (39)	Six items related to early insomnia from the Structured Interview Guide for the Hamilton Depression Rating Scale (SIGH-D)>2	/	Depression (six items) from the brief job stress questionnaire>6	Multiple logistic regression
Vilchez-Cornejo et al. (40)	Designed by researchers: sleep disturbance or not	Number of jobs, sex	MBI-HSS	Multivariate paired logistic regression
Cai et al. (41)	Sleep hours per day	/	PHQ-9>10	Poisson regression
Vidotti et al. (42)	Sleep hours ≤ 6 h per day	Age,Marital status, Occupation, Health related variable Exercise, Regular diet, Health status, Psychiatric symptom, Work related variable Hospital rank, Turnover intention, Physician–patient relationship	The 11-item Chalder fatigue scale (CFS) for measuring burnout >4.	Multiple logistical regression
Wang et al. (43)	Designed by researchers. Continuous variable from 0 (very poor) to10 (perfect)	/	Professional Quality of Life Scale (ProQOL-CN)	Multiple linear regression
Ibrahim et al. (44)	Sleep hours <6 h per day	Age, nationality, exercise, service type, number of meals per day and number of breakfasts	the 21-item Depression Anxiety Stress Scale (DASS-21)>2	Binary and multinomial logistic regression
Dai et al. (45)	PSQI>5	/	HADS>7	Multiple logistic regression
Ghasemi et al. (46)	PSQI	/	Swedish Occupational Fatigue Inventory (SOFI)	Structural equation modelling
**Cross-sectional studies**				
Aydin Sayilan et al. (47)	ISI>2	/	DASS-21	Multivariable logistic regression
Youssef et al. (48)	PSQI	Gender	Post-traumatic Stress Disorder Checklist for DSM-5 (PCL-5) >33	hierarchical multiple regression
Yin et al. (49)	sleep disorder screening	/	MBI-HSS	Not mentioned
Weaver et al. (4)	Sleep hours <6 h per day	Age, study site, alcohol drinking, smoking, exercise, hypnotic medication use, and night shift work	Core symptoms of depression>2	Multivariable logistic regression
Ji et al. (50)	PSQI>7		SAS>50	Pearson linear correlation
Furihata et al. (51)	Sleep hours per day	Demographic and clinical variables and comorbid psychological symptoms	Short health anxiety inventory (SHAI)	Hierarchical multivariable regression
Cheng et al. (52)	Sleep hours per day	/	CBI>50	Multivariate linear regression
Higgins et al. (53)	PSQI>5	/	MBI	Multiple regression analysis
Ng et al. (54)	The PROMIS Sleep Disturbance Short Form	/	PHQ-9	Structural Equation Modelling
Ding et al. (55)	Sleep Habits Questionnaire (CHAS)	/	Goldberg’s General Health Questionnaire (GHQ-28)	Linear regression
Eva et al. (56)	PSQI>5	/	Generalized Anxiety Disorder 7-item scale (GAD-7) ≥ 4	Multivariate logistic regression
Tu et al. (57)	Design by researchers: sleep disturbance or not	/	MBI-HSS	Multiple logistic regression analysis
Magnavita et al. (58)	Sleep Condition Indicator (SCI)	Age, marital status, offspring status, current work tenure, professional status and depression status	PHQ-9: item No.9 >1	Multivariate logistic regression
Korkmaz et al. (59)	PSQI>5	/	Beck Anxiety Inventory (BAI) >7	Pearson correlation
Secosan et al. (60)	PSQI	/	Swedish Occupational Fatigue Inventory (SOFI)	Pearson correlation
Teo et al. (61)	Designed by researchers: good sleep quality or poor sleep quality.	Age, sex	Generalized Anxiety Disorder 7-item (GAD-7)>5. The Zung Self-Rating Depression Scale (SDS)>50	Multivariate logistic regression
Tasdemir et al. (62)	ISI ≥ 8	/	General Health Questionnaire-12 (GHQ-12) > 16	Multiple linear regression
Wang et al. (63)	Sleep duration ≤ 6 h per day/PROMIS Sleep Disturbance Short Form	Social support, work-family conflict, and negative behaviors at work	The 10-item version of the Center for Epidemiologic Studies Depression Scale (CES-D)>10	Multivariate linear regression
Mousavi et al. (64)	PSQI	/	SAS	Spearman correlation
Simonetti et al. (65)	PSQI ≥ 5	/	HADS-Anxiety part >10. HADS-depression part >7.	Spearman Correlation
Zhang et al. (13)	PSQI>7	/	The Impact of Event Scale (IES-R) >33	Multivariate logistic regression
Zhang et al. (66)	ISI>7	/	IES-R>7	Multivariate logistic regression
Yitayih et al. (67)	PSQI	/	CES-D>16	Multivariate logistic regression
Chen et al. (68)	ISI>7	/	DASS-21: depression part>4, anxiety part>3 or stress part>7.	structural equation modelling
Kandemir et al. (69)	ISI	/	The Maslach Burnout Inventory-Short Form (MBI-SF)	Spearman correlation
Aydin et al. (70)	ISI>5	Age, body mass index, sex (if appropriate), physical activity, household income, working time, night shifts, visiting friends constantly, religious or not, marital status, siblings or not, experienced a major life event or not, current year of residency, smoking status, alcohol consumption, coffee intake, and specialty.	PHQ-9>5	Multivariate logistic regression
**Cross-sectional studies**				
Chang et al. (71)	PSQI>5	/	The Link Burnout Questionnaire (LBQ)>0	Linear regression
Mokros et al. (72)	Sleep hours per day	Demographic and clinical variables, and comorbid psychological symptoms.	short health anxiety inventory (SHAI)>27	Multivariate logistic regression
Abdelghani et al. (73)	Sleep quality scale: 0-10 scale (Reverse scoring).	/	GAD-7 for measuring anxiety>10; PHQ-9 for measuring depression>10.	Multivariate logistic regression
Abu-Elenin (74)	Designed by researchers for measuring sleep quality: good/general/bad/very bad	/	GAD-7 for measuring anxiety>10; PHQ-9 for measuring depression>10.	Linear regression
Pang et al. (18)	Designed by researchers:choose sleep satisfaction or sleep deprivation	/	MBI	Multivariate logistic regression
Abbas et al. (77)	PSQI>5	/	12-item General Health Questionnaire>2	Pearson correlation
Olagunju et al. (76)	PSQI>5	/	CES-D>15	Pearson correlation
Hsieh et al. (77)	PSQI>7	/	GAD-7 ≥ 4	Pearson correlation
Jiang et al. (78)	One item extracted out of PSQI	/	PTSD checklist for DSM-5 (PCL-5) ≥ 33	Hierarchical regression analysis
Geng et al. (79)	PSQI ≥ 6	/	PHQ-9>5	Multivariate logistic regression
Garcia et al. (80)	How many nightmares did you have that woke you up?—If the answer was greater than one time, the sleep quality was poor.	/	‘I felt stressed’, rated on a scale of 0 (not at all) to 4 (extremely)	Multilevel models

**FIGURE 2:**
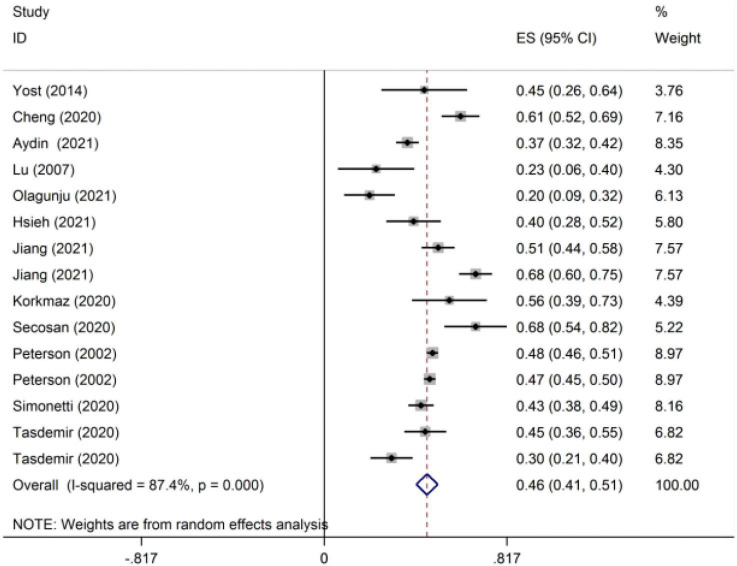
The meta-analytic estimates of the correlation coefficient (r)*. *The data were integrated as Fisher’s Z (Zr).

For studies that reported the correlation coefficient, we combined *r* as the effect indicator. A total of 12 studies were included in the meta-analysis estimates of *r* (Figure 2). Because of the large heterogeneity of the included studies (*I*^2^ = 87.4% > 50%), RE was used to integrate the effect size. The integrated statistic *r* was 0.43, and the 95% CI was 0.39–0.47. The contrast of *r* between the epidemic and non-epidemic periods of COVID-19 is shown in Table 3. The *r* during the epidemic of new coronary pneumonia was 0.45 (95% CI: 0.37–0.53), while the *r* during the non-epidemic period was 0.40 (95% CI: 0.36–0.44). The comparison of the correlation between sleep disorders and different types of mental health is shown in Table 3. The *r* values of burnout, anxiety, depression, distress and mental health complaints were 0.35 (95% CI: 0.31–0.39), 0.46 (95% CI: 0.42–0.49), 0.44 (95% CI: 0.32–0.54), 0.20 (95% CI: 0.09–0.31) and 0.59 (95% CI: 0.49–0.67), respectively.

**TABLE 3:** Subgroup analysis results of the association between sleep disturbance and mental health.

Outcome	Number of included studies	Heterogeneity test results		Effect model	Meta-analysis result	
		*P*	*I*^2^ (%)		Effect size(95%CI)	*P*
*Correlation coefficient*	12	<0.001	87.4	RE^1^	0.43 (0.39–0.47)	<0.001
COVID-19						
Non-epidemic period	6	<0.001	79.0	RE	0.40 (0.36–0.44)	<0.001
Epidemic period	7	<0.001	90.6	RE	0.45 (0.37–0.53)	<0.001
Mental health						
Burnout	3	0.210	35.9	FE	0.35 (0.31–0.39)	<0.001
Anxiety	7	0.022	61.9	RE	0.46 (0.42–0.49)	<0.001
Depression	4	<0.001	93.1	RE	0.44(0.32–0.54)	<0.001
Distress	1	/	/	/	0.20 (0.09–0.31)	<0.001
Mental health complaints	1	/	/	/	0.59 (0.49–0.67)	<0.001
*Odds ratio*	18	<0.001	89.4	RE	3.74 (2.76–5.07)	<0.001
COVID-19						
Non-epidemic period	11	<0.001	88.3	RE	3.18 (2.08–4.85)	<0.001
Epidemic period	7	<0.001	91.1	RE	4.48 (2.74–7.32)	<0.001
Mental health						
PTSD	1	/	/	/	5.70 (2.89–11.23)	<0.001
Anxiety	3	0.006	80.4	RE	3.57 (1.19–10.74)	0.023
Depression	10	<0.001	93.6	RE	3.24 (2.10–5.01)	<0.001
Stress	2	0.040	76.3	RE	8.91 (2.54–31.28)	<0.001
Burnout	6	0.299	17.6	FE	3.20 (2.34–4.37)	<0.001
Sleep disturbance						
Low sleep quality	14	<0.001	89.8	RE	4.08 (2.86–5.81)	<0.001
Short sleep duration	4	<0.001	80.7	RE	2.66 (1.51–4.67)	<0.001

^1^RE, random effect model; FE, fixed effect model.

**FIGURE 3:**
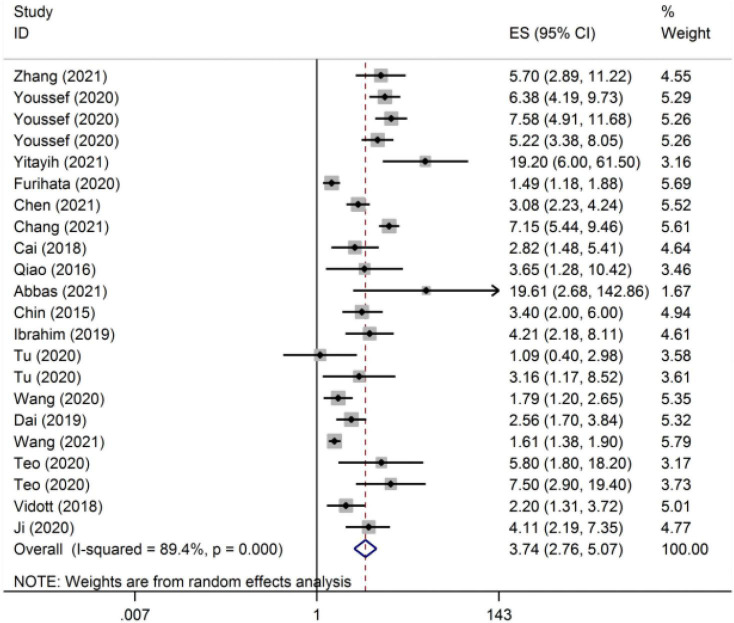
The meta-analytic estimates of the odds ratio (OR).

We used OR as the effect indicator for studies that reported β and OR. The meta-analysis estimate of the OR is shown in Figure 3. We chose the random effect model to combine the effect sizes resulting from *I*^2^ > 50%. The overall OR was 3.74 (95% CI: 2.76–5.07). Subgroup analysis compared the OR results in epidemic and non-epidemic periods of COVID-19, which were 4.48 (95% CI: 2.75–5.07) and 3.74 (95% CI: 2.74–7.32), respectively (Table 3). In the different subgroups of mental health types, the OR was also different (Table 3). The OR of stress was the highest (8.91, 95% CI: 2.54–31.28). Subgroup analysis compared the OR results in epidemic and non-epidemic periods of COVID-19, which were 4.48 (95% CI: 2.75–5.07) and 3.74 (95% CI: 2.74–7.32), respectively.

### Sensitivity analysis

**FIGURE 4:**
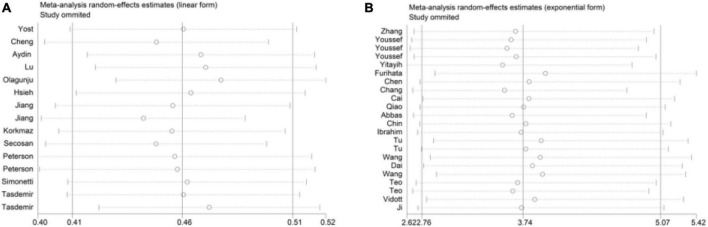
Sensitivity analysis. **(A)** The studies were extracted. **(B)** The studies were extracted OR.

In this meta-analysis, each study was randomly removed to verify the sensitivity. Sensitivity analysis showed that the results of this study were stable (Figure 4).

### Publication bias analysis

**FIGURE 5:**
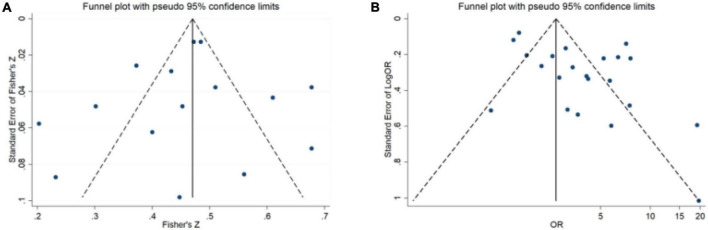
Publication bias analysis. **(A)** The studies were extracted. **(B)** The studies were extracted OR.

The funnel plot of the correlation coefficient between sleep disturbance and mental health demonstrated a symmetrical distribution, indicating that there was no obvious publication bias (Figure 5A). However, the funnel plot demonstrated an asymmetrical distribution of the studies that reported ORs, indicating that there may be publication bias (Figure 5B).

## Discussion

To the best of our knowledge, this meta-analysis was the first to examine the association between sleep disturbance and the mental health of HCWs. At the same time, the subgroup analysis discussed the relationship between different types of sleep disturbance and mental health, as well as sleep disturbance and different types of mental health problems. Moreover, it contrasted sleep disturbance and mental health during the COVID-19 pandemic with the non-COVID-19 pandemic. We found that the sleep disturbance level was positively correlated with mental health among HCWs. In other words, the more serious the sleep disorder, the more serious the mental health problems would be. HCWs with sleep problems have a 3.74 higher risk of mental health problems. The *r* during the COVID-19 epidemic was 0.46 (95% CI: 0.41–0.51), while it was 0.43 (95% CI 0.38–0.48) during the non-epidemic period. The ORs for the epidemic and non-epidemic periods of COVID-19 were 4.48 (95% CI: 2.75–5.07) and 3.74 (95% CI: 2.74–7.32), respectively. This reminded us of the importance of sleep problems among HCWs.

### Quality evaluation of included studies

In the quality evaluation of these studies, 58.18% of the studies were of medium or low quality. Only 41.82% of the literature was high quality. In addition, the quantitative analysis of this study found that the heterogeneity of the study was large. This may be caused by the different questionnaires used in the included studies. The questionnaires for evaluating sleep disturbance were the PSQI and ISI, which have good reliability and validity. However, it seems necessary to choose an exact cut-off value for sleep disturbance in the face of the same population, for example, HCWs. On the one hand, it is conducive to the consistency of our judgment of the overall sleep status of HCWs. On the other hand, it further increases the uniformity of different research results. Many types of questionnaires on mental health appeared in the included studies. Some questionnaires have been used for patients (78). Whether it can be applied to HCWs remains to be determined. The evaluation items designed by the researchers themselves should also be used after testing their reliability and validity in the research. However, this part of the explanation was missing in the research we included (18, 31, 32, 42, 43, 50, 61, 75).

Regrettably, there were only four prospective studies in this meta-analysis, including one cohort study and three longitudinal studies. Due to the small number of prospective studies, the interpretation of the study results on the relationship between sleep and mental health was limited. Therefore, more high-quality prospective studies are still needed in the future.

### Association between sleep disturbance and mental health

This study found that sleep disturbance level was positively correlated with mental health problems among HCWs, of whom sleep disturbance was associated with a higher risk of mental health problems. This was like the results of (81, 82). Children with sleep disorders were 1.5 times more likely to have depression symptoms than children without sleep disorders (95% CI: 1.13–2.00). O’Callaghan’s meta-analysis result between subjective sleep and depressive symptoms among adolescents showed that *r* was 0.41, which was like the results of our study (82).

Recently, studies on the association between sleep disturbance and mental health have proliferated. Most studies were based on certain types of patients or healthy people (83, 84), but studies focusing on HCWs are rare. Some previous studies believe that the relationship between the two is mutual; that is, sleep disorders and mental health affect each other (85). However, there is still a lack of a unified theory and mechanism to explain the relationship. A review of studies pointed out that the underlying mechanisms include the theory of monoamine disorders, the theory of stress response change, the theory of immune system response, and the theory of neurotrophy (86). For example, the dysregulation of monoamine production and delivery is still considered to be an important factor in regulating mood, emotion, cognition, motivational behavior and stress response (87). In experimental studies of chronic sleep deprivation in animals, a reduction in monoamine production has been shown, including norepinephrine and dopamine (88). Due to the decrease in the production of monoamines in the serum, the individual’s vulnerability to mood changes leads to mental health problems (89). This provides a theoretical basis for the positive correlation between sleep disturbance and mental health. However, these mechanisms are still controversial. Therefore, future research should deeply explore the in-depth mechanism of the association between sleep disturbance and mental health.

Sleep disturbance was more closely related to mental health during the COVID-19 epidemic. HCWs with sleep disturbance had a 4.48 (95% CI: 2.74–7.32) times higher risk of mental health problems than those without sleep disorders. This result was like the result of Naglaa et al. (48). The reason may be that during the COVID-19 pandemic, HCWs had to be under long-term work-time (48). The working environment was more complex, the daily workload was greater, and the need for sleep was also higher (61). To date, with the continual state of the COVID-19 pandemic, HCWs around the world are still facing huge challenges, including a higher risk of infection, fears and worries about the epidemic, excessive work patterns, long-term isolation, coping with the patient’s negative emotions and lack of family contact (49). These factors will affect their sleep status. In addition, they were in a state of high arousal more potentially, which will seriously affect sleep (49). Kandemir et al. investigated 192 frontline HCWs in the fight against the epidemic and found that approximately 80.41% of them had sleep disturbance (69). Therefore, psychological problems were more likely to occur when sleep disturbance occurred.

In the subgroup analysis, we found that both low sleep quality and short sleep duration increased the risk of mental health problems for medical staff. Research by Furihata et al. also found that short sleep duration and poor sleep quality increase the risk of depression (51). The reason may be that poor sleep quality will increase the worry of medical staff and lead to adverse mental health events (35). Due to the short sleep duration, on the one hand, the integrity of the sleep cycle cannot be guaranteed. On the other hand, the rapid eye movement cycle in the sleep cycle may be shortened. The period of monoamine secretion is concentrated in the rapid eye movement cycle according to the theory of monoamine disorders (90). Subsequently, the reduction in serum monoamines affects the individual’s mental health.

Based on the above findings, we suggest that HCWs should put emphasis on sleep disturbance, including short duration and low quality. Actions should be taken as early as possible when the impact of sleep disturbance on individuals and lives is detected. For example, HCWs can take sleep-promoting drugs early or find ways to help them get better sleep. Of course, these methods may vary from person to person. So there is demanding for more RCT studies to verify effective measures to help HCWs to promote sleep in the future. Furthermore, administrators should allocate human resources rationally to balance individual workloads of HCWs. At the same time, it will helpful to alleviate sleep disturbance through flexible scheduling mechanism to ensure that HCWs have sufficient sleep time and rest time.

### Strengths and limitations

Our study has several advantages. First, the number of studies included in this review was large. The subjects of the study were HCWs, which is an important part of the health system. In the results section, the patients were divided into multiple subgroups for analysis. The results of this study laid the foundation for the subsequent formulation of interventions. For related departments, it is possible to improve the mental health of medical workers by intervening in their sleep quality or increasing their sleep time.

Nevertheless, there were still some limitations. First, we can see that this meta-analysis has highly heterogeneous. Additionally, there were few prospective studies. Therefore, there was a lack of cohort studies that can directly verify the causal relationship between sleep disturbance and mental health. In the future, more cohort studies still need to be included to further demonstrate the causal relationship between sleep disturbance and the mental health of HCWs. Third, due to the limited data defining sleep disturbance as short sleep duration, a subgroup analysis of the correlation between different sleep disturbance types and mental health was not carried out. Fourth, only literature published in English was included. Finally, only the Web of Science, PubMed, EMBASE, CINAHL, Psyclnfo, and Cochrane Library databases were searched for analyses. Hence, there may be literature that was not retrieved, which may have a potential influence on the results.

## Conclusion

In summary, this meta-analysis mainly explored the association between sleep disturbance and the mental health of HCWs. It included 59 original studies for qualitative analysis and 30 of them for quantitative analysis. Meanwhile, sleep disturbance and mental health problems were positively correlated among HCWs. Sleep disturbance increased the risk of mental health problems. Moreover, subgroup analysis showed that sleep disturbance and mental health problems were higher during the COVID-19 pandemic. The risk of mental health problems was also elevated during this period. Therefore, the public should keep a watchful eye on HCWs’ sleep problems. Particularly in the COVID-19 epidemic today, more attention should be given to this issue.

## Author contributions

YL designed and conducted information retrieval, risk of bias assessment, and wrote the manuscript. YL, HZ, and FJ assisted in searching the database and extracted and reviewed the data. LH and QZ finished the statistical analysis. HC and YZ supervised the design, reviewed the results, and revised the manuscript. All authors listed meet the author eligibility criteria according to the latest guidelines of the International Committee of Medical Journal Editors (ICMJE). All authors agreed with the manuscript.
