# Supplementary material for: Association between sleep disturbance and mental health of healthcare workers: A systematic review and meta-analysis
Source: Front Psychiatry. 2022 Jul 29;13:919176. doi: 10.3389/fpsyt.2022.919176 (PMC9372625; doi:10.3389/fpsyt.2022.919176)
Supplement: Supplementary file 1 [file Data_Sheet_1.docx]

Supplementary Material

**Appendix 1 retrieval records**

- **Retrieve records from Web of Science**

4 ((#3) AND #2) AND #1 2,651

3 ((((((((((((((((((((((TI=(Health Care Provider)) OR TI=(Health worker)) OR TI=(Healthcare Provider)) OR TI=(Healthcare Worker)) OR TI=(Medical worker)) OR TI=(Nurse)) OR TI=(Physician)) OR TI=(Health Personnel)) OR TI=(Delivery of Health Care)) OR TI=(Medical staff)) OR TI=(Doctor)) OR TI=(hospital worker)) OR TI=(Healthcare)) OR TI=(paramedic)) OR TI=(Physical therapist)) OR TI=(therapist)) OR TI=(Caregiver)) OR TI=(Clinic)) OR TI=(Allied Health Personnel)) OR TI=(health professional)) OR TI=(healthcare professional)) OR TI=(Medic)) OR TI=(Nursing Assistant) 495,716

2 ((((((((((TS=(Mental health)) OR TS=(mental disorders)) OR TS=(melancholi)) OR TS=(anxiety)) OR TS=(mood disorders)) OR TS=(depression)) OR TS=(stress)) OR TS=(distress)) OR TS=(emotion)) OR TS=(burnout)) OR TS=(Stress, Psychological) 3,236,299

1 ((((((((TS=(Sleep )) OR TS=(dream )) OR TS=(nightmare)) OR TS=(parasomnia)) OR TS=(insomnia)) OR TS=(circadian)) OR TS=(dyssomni)) OR TS=(chronotype)) OR TS=(Sleep Wake Disorders) 389488

- **Retrieve records from EMBASE (via OVID)**

**Embase <1974 to 2021 August 16>**

1 exp sleep/ 251402

2 dream.ab. or dream.ti. 9504

3 Sleep.ab. or Sleep.ti. 271800

4 nightmare.ab. or nightmare.ti. 2198

5 parasomnia.ab. or parasomnia.ti. 1262

6 insomnia.ab. or insomnia.ti. 39777

7 Dyssomnia.ab. or Dyssomnia.ti. 117

8 circadian.ab. or circadian.ti. 65733

9 chronotype.ab. or chronotype.ti. 2242

10 Sleep Wake Disorders.ab. or Sleep Wake Disorders.ti. 393

11 1 or 2 or 3 or 4 or 5 or 6 or 7 or 8 or 9 or 10 436920

12 exp mental health/ 179458

13 exp mental stress/ 88248

14 Melancholi.ab. or Melancholi.ti. 0

15 Mental health.ab. or Mental health.ti. 204830

16 mental disorders.ab. or mental disorders.ti. 44248

17 anxiety.ab. or anxiety.ti. 302294

18 mood disorders.ab. or mood disorders.ti. 21609

19 depression.ab. or depression.ti. 495092

20 stress.ab. or stress.ti. 1013912

21 distress.ab. or distress.ti. 176430

22 emotion.ab. or emotion.ti. 52257

23 burnout.ab. or burnout.ti. 17784

24 Stress, Psychological.ab. or Stress, Psychological.ti. 336

25 12 or 13 or 14 or 15 or 16 or 17 or 18 or 19 or 20 or 21 or 22 or 23 or 24 2002073

26 exp health care personnel/ 1692025

27 exp health care personnel/ 1692025

28 exp health care delivery/ 3561014

29 exp medical staff/ 38256

30 Health Care Provider.ab. or Health Care Provider.ti. 8505

31 Health worker.ab. or Health worker.ti. 4346

32 Healthcare Provider.ab. or Healthcare Provider.ti. 6591

33 Healthcare Worker.ab. or Healthcare Worker.ti. 2055

34 Medical worker.ab. or Medical worker.ti. 110

35 Nurse.ab. or Nurse.ti. 149951

36 Physician.ab. or Physician.ti. 271466

37 Health Personnel.ab. or Health Personnel.ti. 5116

38 Medical staff.ab. or Medical staff.ti. 19400

39 Doctor.ab. or Doctor.ti. 76889

40 paramedic.ab. or paramedic.ti. 4070

41 26 or 27 or 28 or 29 or 30 or 31 or 32 or 33 or 34 or 35 or 36 or 37 or 38 or 39 or 40 4993769

42 11 and 25 and 41 21131

43 limit 42 to article 10118

44 limit 43 to full text 3167

- **Retrieve records from PubMed**

sleep:

Search: (((((((((((Sleep[MeSH Terms]) OR (Sleep[Title/Abstract])) OR (Sleep Wake Disorders[Title/Abstract])) OR (Sleep Wake Disorders[MeSH Terms])) OR (Sleep[Title/Abstract])) OR (dream[Title/Abstract])) OR (nightmare[Title/Abstract])) OR (parasomnia[Title/Abstract])) OR (insomnia[Title/Abstract])) OR (circadian[Title/Abstract])) OR (dyssomni[Title/Abstract])) OR (chronotype[Title/Abstract])

Mental health:

Search: ((((((((((((((((Mental health[MeSH Terms]) OR (Mental health[Title/Abstract])) OR (mental disorders[Title/Abstract])) OR (mental disorders[MeSH Terms])) OR (anxiety[Title/Abstract])) OR (anxiety[MeSH Terms])) OR (depression[MeSH Terms])) OR (depression[Title/Abstract])) OR (Stress, Psychological[Title/Abstract])) OR (Stress, Psychological[MeSH Terms])) OR (Stress, Psychological[Title/Abstract])) OR (melancholi[Title/Abstract])) OR (mood disorders[Title/Abstract])) OR (stress[Title/Abstract])) OR (distress[Title/Abstract])) OR (emotion[Title/Abstract])) OR (burnout[Title/Abstract])

Healthcare worker

Search: ((((((((((((Health Personnel[MeSH Terms]) OR (Health Personnel[Title/Abstract])) OR (Delivery of Health Care[Title/Abstract])) OR (Delivery of Health Care[MeSH Terms])) OR (Medical staff[MeSH Terms])) OR (Medical staff[Title/Abstract])) OR (Health Care Provider[Title/Abstract])) OR (Health worker[Title/Abstract])) OR (Healthcare Provider[Title/Abstract])) OR (Healthcare Worker[Title/Abstract])) OR (Medical worker[Title/Abstract])) OR (Nurse[Title/Abstract])) OR (Physician[Title/Abstract])

ALL：—— result：8475

Search: ((((((((((((((Health Personnel[MeSH Terms]) OR (Health Personnel[Title/Abstract])) OR (Delivery of Health Care[Title/Abstract])) OR (Delivery of Health Care[MeSH Terms])) OR (Medical staff[MeSH Terms])) OR (Medical staff[Title/Abstract])) OR (Health Care Provider[Title/Abstract])) OR (Health worker[Title/Abstract])) OR (Healthcare Provider[Title/Abstract])) OR (Healthcare Worker[Title/Abstract])) OR (Medical worker[Title/Abstract])) OR (Nurse[Title/Abstract])) OR (Physician[Title/Abstract])) AND (((((((((((((((((Mental health[MeSH Terms]) OR (Mental health[Title/Abstract])) OR (mental disorders[Title/Abstract])) OR (mental disorders[MeSH Terms])) OR (anxiety[Title/Abstract])) OR (anxiety[MeSH Terms])) OR (depression[MeSH Terms])) OR (depression[Title/Abstract])) OR (Stress, Psychological[Title/Abstract])) OR (Stress, Psychological[MeSH Terms])) OR (Stress, Psychological[Title/Abstract])) OR (melancholi[Title/Abstract])) OR (mood disorders[Title/Abstract])) OR (stress[Title/Abstract])) OR (distress[Title/Abstract])) OR (emotion[Title/Abstract])) OR (burnout[Title/Abstract]))) AND ((((((((((((Sleep[MeSH Terms]) OR (Sleep[Title/Abstract])) OR (Sleep Wake Disorders[Title/Abstract])) OR (Sleep Wake Disorders[MeSH Terms])) OR (Sleep[Title/Abstract])) OR (dream[Title/Abstract])) OR (nightmare[Title/Abstract])) OR (parasomnia[Title/Abstract])) OR (insomnia[Title/Abstract])) OR (circadian[Title/Abstract])) OR (dyssomni[Title/Abstract])) OR (chronotype[Title/Abstract]))

After reset filter：-1975

**((((((((((((((Health Personnel[MeSH Terms]) OR (Health Personnel[Title/Abstract])) OR (Delivery of Health Care[Title/Abstract])) OR (Delivery of Health Care[MeSH Terms])) OR (Medical staff[MeSH Terms])) OR (Medical staff[Title/Abstract])) OR (Health Care Provider[Title/Abstract])) OR (Health worker[Title/Abstract])) OR (Healthcare Provider[Title/Abstract])) OR (Healthcare Worker[Title/Abstract])) OR (Medical worker[Title/Abstract])) OR (Nurse[Title/Abstract])) OR (Physician[Title/Abstract])) AND (((((((((((((((((Mental health[MeSH Terms]) OR (Mental health[Title/Abstract])) OR (mental disorders[Title/Abstract])) OR (mental disorders[MeSH Terms])) OR (anxiety[Title/Abstract])) OR (anxiety[MeSH Terms])) OR (depression[MeSH Terms])) OR (depression[Title/Abstract])) OR (Stress, Psychological[Title/Abstract])) OR (Stress, Psychological[MeSH Terms])) OR (Stress, Psychological[Title/Abstract])) OR (melancholi[Title/Abstract])) OR (mood disorders[Title/Abstract])) OR (stress[Title/Abstract])) OR (distress[Title/Abstract])) OR (emotion[Title/Abstract])) OR (burnout[Title/Abstract]))) AND ((((((((((((Sleep[MeSH Terms]) OR (Sleep[Title/Abstract])) OR (Sleep Wake Disorders[Title/Abstract])) OR (Sleep Wake Disorders[MeSH Terms])) OR (Sleep[Title/Abstract])) OR (dream[Title/Abstract])) OR (nightmare[Title/Abstract])) OR (parasomnia[Title/Abstract])) OR (insomnia[Title/Abstract])) OR (circadian[Title/Abstract])) OR (dyssomni[Title/Abstract])) OR (chronotype[Title/Abstract])) Filters: Clinical Study, Clinical Trial, Clinical Trial, Phase I, Clinical Trial, Phase II, Clinical Trial, Phase III, Clinical Trial, Phase IV, Clinical Trial, Veterinary, Comparative Study, Controlled Clinical Trial, Editorial, Letter, Multicenter Study, Observational Study, Randomized Controlled Trial, Observational Study, Veterinary**

**Retrieve records from Cochrane Library (via OVID)**

**EBM Reviews - Cochrane Central Register of Controlled Trials <July 2021>**

**EBM Reviews - Cochrane Database of Systematic Reviews <2005 to August 18, 2021>**

**1 exp sleep/ 5949**

**2 dream.ab. or dream.ti. 495**

**3 Sleep.ab. or Sleep.ti. 37214**

**4 nightmare.ab. or nightmare.ti. 143**

**5 parasomnia.ab. or parasomnia.ti. 34**

**6 insomnia.ab. or insomnia.ti. 8592**

**7 circadian.ab. or circadian.ti. 3486**

**8 Dyssomnia.ab. or Dyssomnia.ti. 18**

**9 chronotype.ab. or chronotype.ti. 178**

**10 Sleep Wake Disorders.ab. or Sleep Wake Disorders.ti. 14**

**11 1 or 2 or 3 or 4 or 5 or 6 or 7 or 8 or 9 or 10 44277**

**12 exp mental health/ 1679**

**13 exp mental disorders/ 72901**

**14 exp anxiety/ 8359**

**15 exp depression/ 13002**

**16 exp stress, psychological/ 6312**

**17 Melancholi.ab. or Melancholi.ti. 0**

**18 Mental health.ab. or Mental health.ti. 19362**

**19 mental disorders.ab. or mental disorders.ti. 4798**

**20 anxiety.ab. or anxiety.ti. 50928**

**21 mood disorders.ab. or mood disorders.ti. 1274**

**22 depression.ab. or depression.ti. 73111**

**23 stress.ab. or stress.ti. 55410**

**24 distress.ab. or distress.ti. 21012**

**25 emotion.ab. or emotion.ti. 5580**

**26 burnout.ab. or burnout.ti. 1047**

**27 Stress, Psychological.ab. or Stress, Psychological.ti. 146**

**28 12 or 13 or 14 or 15 or 16 or 17 or 18 or 19 or 20 or 21 or 22 or 23 or 24 or 25 or 26 or 27 217289**

**29 exp health personnel/ 9124**

**30 exp "Delivery of Health Care"/ 45294**

**31 exp medical staff/ 343**

**32 Health Care Provider.ab. or Health Care Provider.ti. 730**

**33 Health worker.ab. or Health worker.ti. 1046**

**34 Healthcare Provider.ab. or Healthcare Provider.ti. 642**

**35 Healthcare Worker.ab. or Healthcare Worker.ti. 151**

**36 Medical worker.ab. or Medical worker.ti. 3**

**37 Nurse.ab. or Nurse.ti. 16375**

**38 Physician.ab. or Physician.ti. 26415**

**39 Health Personnel.ab. or Health Personnel.ti. 195**

**40 Delivery of Health Care.ab. or Delivery of Health Care.ti. 50**

**41 Medical staff.ab. or Medical staff.ti. 1041**

**42 paramedic.ab. or paramedic.ti. 529**

**43 29 or 30 or 31 or 32 or 33 or 34 or 35 or 36 or 37 or 38 or 39 or 40 or 41 or 42 89539**

**44 11 and 28 and 43 1177**

- **Retrieve records from Psyclnfo database**

| **#** | **Query** | **Limiters/Expanders** | **Last Run Via** | **Results** |
| --- | --- | --- | --- | --- |
| S15 | S5 AND S11 AND S14 | Expanders - Apply equivalent subjects Search modes - Boolean/Phrase | Interface - EBSCOhost Research Databases Search Screen - Advanced Search Database - APA PsycInfo | 29 |
| S14 | S12 OR S13 | Expanders - Apply equivalent subjects Search modes - Boolean/Phrase | Interface - EBSCOhost Research Databases Search Screen - Advanced Search Database - APA PsycInfo | 90,254 |
| S13 | KW hospital worker OR KW Healthcare OR KW paramedic OR KW Physical therapist OR KW therapist OR KW Caregiver OR KW Clinic OR KW Allied Health Personnel OR KW health professional OR KW healthcare professional OR KW Medic OR KW Nursing Assistant | Expanders - Apply equivalent subjects Search modes - Boolean/Phrase | Interface - EBSCOhost Research Databases Search Screen - Advanced Search Database - APA PsycInfo | 39,669 |
| S12 | MA Health Personnel OR KW Delivery of Health Care OR KW Medical staff OR KW Health Care Provider OR KW Healthcare Worker OR KW Medical worker OR KW Nurse OR KW Physician OR KW Health Personnel OR KW Delivery of Health Care OR KW Medical staff OR KW Doctor | Expanders - Apply equivalent subjects Search modes - Boolean/Phrase | Interface - EBSCOhost Research Databases Search Screen - Advanced Search Database - APA PsycInfo | 53,226 |
| S11 | S9 OR S10 | Expanders - Apply equivalent subjects Search modes - Boolean/Phrase | Interface - EBSCOhost Research Databases Search Screen - Advanced Search Database - APA PsycInfo | 42,551 |
| S10 | S3 AND S8 | Limiters - Publication Type: All Journals; Exclude Dissertations Expanders - Apply equivalent subjects Search modes - Boolean/Phrase | Interface - EBSCOhost Research Databases Search Screen - Advanced Search Database - APA PsycInfo | 7,345 |
| S9 | S6 OR S7 | Limiters - Publication Type: All Journals; Methodology: -Longitudinal Study; Exclude Dissertations Expanders - Apply equivalent subjects Search modes - Boolean/Phrase | Interface - EBSCOhost Research Databases Search Screen - Advanced Search Database - APA PsycInfo | 35,661 |
| S8 | S6 OR S7 | Expanders - Apply equivalent subjects Search modes - Boolean/Phrase | Interface - EBSCOhost Research Databases Search Screen - Advanced Search Database - APA PsycInfo | 514,792 |
| S7 | KW distress OR KW emotion OR KW burnout OR Stress, Psychological | Limiters - Publication Type: All Journals; Exclude Dissertations Expanders - Apply equivalent subjects Search modes - Boolean/Phrase | Interface - EBSCOhost Research Databases Search Screen - Advanced Search Database - APA PsycInfo | 109,179 |
| S6 | MA Mental health OR MA mental disorders OR MA anxiety OR MA depression OR MA Stress, Psychological OR KW Melancholi* OR KW Mental health OR KW mental disorders OR KW anxiety OR KW mood disorders OR KW depression OR KW stress | Limiters - Publication Type: All Journals; Exclude Dissertations Expanders - Apply equivalent subjects Search modes - Boolean/Phrase | Interface - EBSCOhost Research Databases Search Screen - Advanced Search Database - APA PsycInfo | 468,936 |
| S5 | S1 OR S4 | Expanders - Apply equivalent subjects Search modes - Boolean/Phrase | Interface - EBSCOhost Research Databases Search Screen - Advanced Search Database - APA PsycInfo | 4,913 |
| S4 | S2 AND S3 | Expanders - Apply equivalent subjects Search modes - Boolean/Phrase | Interface - EBSCOhost Research Databases Search Screen - Advanced Search Database - APA PsycInfo | 1,097 |
| S3 | “cross-sectional study” | Limiters - Publication Type: All Journals; Exclude Dissertations Expanders - Apply equivalent subjects Search modes - Boolean/Phrase | Interface - EBSCOhost Research Databases Search Screen - Advanced Search Database - APA PsycInfo | 27,180 |
| S2 | MA Sleep OR MA Sleep Wake Disorders OR KW Sleep OR KW dream OR KW nightmare OR KW parasomnia OR KW insomnia OR KW circadian OR KW Dyssomnia OR KW chronotype OR KW Sleep Wake Disorders OR KW Sleep Wake Disorders | Limiters - Publication Type: All Journals; Exclude Dissertations Expanders - Apply equivalent subjects Search modes - Boolean/Phrase | Interface - EBSCOhost Research Databases Search Screen - Advanced Search Database - APA PsycInfo | 65,340 |
| S1 | MA Sleep OR MA Sleep Wake Disorders OR KW Sleep OR KW dream OR KW nightmare OR KW parasomnia OR KW insomnia OR KW circadian OR KW Dyssomnia OR KW chronotype OR KW Sleep Wake Disorders OR KW Sleep Wake Disorders | Limiters - Publication Type: All Journals; Methodology: -Longitudinal Study; Exclude Dissertations Expanders - Apply equivalent subjects Search modes - Boolean/Phrase | Interface - EBSCOhost Research Databases Search Screen - Advanced Search Database - APA PsycInfo | 3,906 |

- **Retrieve records from CINAHL (via EBSCO)**

**# Query Limiters/Expanders Last Run Via Results**

S19 S6 AND S7 AND S12 AND S18 Expanders - Apply related words; Also

search within the full text of the articles; Apply equivalent subjects Search modes - Boolean/Phrase

Interface - EBSCOhost Research Databases Search Screen - Advanced Search Database - CINAHL

407

S18 S13 OR S14 OR S15 OR S16 OR S17

Expanders - Apply related words; Also search within the full text of the articles; Apply equivalent subjects Search modes - Boolean/Phrase

Interface - EBSCOhost Research Databases Search Screen - Advanced Search Database - CINAHL

489,255

S17 AB Health Care Provider Limiters - Research Article

Expanders - Apply related words; Also search within the full text of the articles; Apply equivalent subjects Search modes - Boolean/Phrase

Interface - EBSCOhost Research Databases Search Screen - Advanced Search Database - CINAHL

23,760

S16 AB Healthcare OR AB hospital worker OR AB Doctor OR AB Medical staff OR AB Delivery of Health Care OR AB Health Personnel OR AB Physician OR AB Nurse OR AB Medical worker OR AB Healthcare Worker OR AB Healthcare Provider OR AB Health worker

Limiters - Research Article

Expanders - Apply related words; Also search within the full text of the articles; Apply equivalent subjects Search modes - Boolean/Phrase

Interface - EBSCOhost Research Databases Search Screen - Advanced Search Database - CINAHL

328,812

S15 TI Medic OR TI Nursing Assistant OR AB Nursing Assistant OR AB Medic OR AB healthcare professional OR

Limiters - Research Article

Expanders - Apply related words; Also search within the full text of the

Interface - EBSCOhost Research Databases Search Screen - Advanced Search Database - CINAHL

170,232

AB health professional OR AB Allied Health Personnel OR AB Clinic OR AB Caregiver OR AB therapist OR AB Physical therapist OR AB paramedic

articles; Apply equivalent subjects Search modes - Boolean/Phrase

S14 TI Medical staff OR TI Doctor OR TI hospital worker OR TI Healthcare OR TI paramedic OR TI Physical therapist OR TI therapist OR TI Caregiver OR TI Clinic OR TI Allied Health Personnel OR TI health professional OR TI healthcare professional

Limiters - Research Article

Expanders - Apply related words; Also search within the full text of the articles; Apply equivalent subjects Search modes - Boolean/Phrase

Interface - EBSCOhost Research Databases Search Screen - Advanced Search Database - CINAHL

79,329

S13 MW Health Personnel OR MW Delivery of Health Care OR MW Medical staff OR TI Health Care Provider OR TI Health worker OR TI Healthcare Provider OR TI Healthcare Worker OR TI Medical worker OR TI Nurse OR TI Physician OR TI Health Personnel OR TI Delivery of Health Care

Limiters - Research Article

Expanders - Apply related words; Also search within the full text of the articles; Apply equivalent subjects Search modes - Boolean/Phrase

Interface - EBSCOhost Research Databases Search Screen - Advanced Search Database - CINAHL

122,008

S12 S9 OR S10 OR S11 Expanders - Apply related words; Also search within the full text of the articles; Apply equivalent subjects Search modes - Boolean/Phrase

Interface - EBSCOhost Research Databases Search Screen - Advanced Search Database - CINAHL

347,852

S11 AB mental disorders OR AB Mental health OR AB Melancholi*

Limiters - Research Article

Expanders - Apply related words; Also search within the full text of the articles; Apply equivalent subjects Search modes - Boolean/Phrase

Interface - EBSCOhost Research Databases Search Screen - Advanced Search Database - CINAHL

73,927

S10 TI distress OR TI emotion OR TI burnout OR TI Stress, Psychological

Limiters - Research Article

Expanders - Apply related words; Also

Interface - EBSCOhost Research Databases Search Screen - Advanced Search

234,942

OR AB Stress, Psychological OR AB burnout OR AB emotion OR AB distress OR AB stress OR AB depression OR AB mood disorders OR AB anxiety

search within the full text of the articles; Apply equivalent subjects Search modes - Boolean/Phrase

Database - CINAHL

S9 MW Mental health OR MW mental disorders OR MW anxiety OR MW depression OR MW Stress, Psychological OR TI Melancholi* OR TI Mental health OR TI mental disorders OR TI anxiety OR TI mood disorders OR TI depression OR TI stress

Limiters - Research Article

Expanders - Apply related words; Also search within the full text of the articles; Apply equivalent subjects Search modes - Boolean/Phrase

Interface - EBSCOhost Research Databases Search Screen - Advanced Search Database - CINAHL

220,942

S8 S6 AND S7 Expanders - Apply related words; Also search within the full text of the articles; Apply equivalent subjects Search modes - Boolean/Phrase

Interface - EBSCOhost Research Databases Search Screen - Advanced Search Database - CINAHL

3,855

S7 S1 OR S2 Expanders - Apply related words; Also search within the full text of the articles; Apply equivalent subjects Search modes - Boolean/Phrase

Interface - EBSCOhost Research Databases Search Screen - Advanced Search Database - CINAHL

47,203

S6 S3 OR S4 Expanders - Apply related words; Also search within the full text of the articles; Apply equivalent subjects Search modes - Boolean/Phrase

Interface - EBSCOhost Research Databases Search Screen - Advanced Search Database - CINAHL

172,007

S5 MW "Cohort study" OR AB "Cohort study" OR TI "Cohort study"

Expanders - Apply related words; Apply equivalent subjects Search modes - Boolean/Phrase

Interface - EBSCOhost Research Databases Search Screen - Advanced Search Database - CINAHL

95,341

S4 TI "Cohort study" OR AB "cohort study" OR MW "Cohort study"

Expanders - Apply related words; Also search within the full text of the

Interface - EBSCOhost Research Databases Search Screen - Advanced Search

95,341

articles; Apply equivalent subjects Search modes - Boolean/Phrase

Database - CINAHL

S3 TI "cross-sectional study" OR AB "cross-sectional study" OR MW "cross-sectional study"

Expanders - Apply related words; Also search within the full text of the articles; Apply equivalent subjects Search modes - Boolean/Phrase

Interface - EBSCOhost Research Databases Search Screen - Advanced Search Database - CINAHL

77,408

S2 TI Sleep OR TI dream OR TI nightmare OR TI parasomnia OR TI insomnia OR TI circadian OR TI Dyssomnia* OR TI chronotype OR TI Sleep Wake Disorders

S1 MW Sleep OR MW Sleep Wake Disorders OR AB Sleep OR AB Sleep Wake Disorders OR AB dream OR AB nightmare OR AB parasomnia OR AB insomnia OR AB circadian OR AB Dyssomnia* OR AB chronotype

Limiters - Research Article

Expanders - Apply related words; Also search within the full text of the articles; Apply equivalent subjects Search modes - Boolean/Phrase

Limiters - Research ArticleExpanders - Apply related words; Also search within the full text of the articles; Apply equivalent subjects Search modes - Boolean/Phrase

Interface - EBSCOhost Research Databases Search Screen - Advanced Search Database - CINAHL

Interface - EBSCOhost Research Databases Search Screen - Advanced Search Database - CINAHL 22,98

46,138

**Appendix 2 risk of bias evaluation**

**Table1 The result of risk bias evaluation about studies (cross-sectional studies)**

| Included studies | **The result of risk bias evaluation** | | | | | | | | | | | |
| --- | --- | --- | --- | --- | --- | --- | --- | --- | --- | --- | --- | --- |
|  | - 1 | - 2 | - 3 | - 4 | - 5 | - 6 | - 7 | - 8 | - 9 | - 10 | - 11* | - Score |
| Zhang Y(13) | 1 | 1 | 1 | unclear | 1 | 0 | 0 | 1 | 0 | 1 | unclear | 6 |
| Zhang R (66) | 1 | 1 | 1 | unclear | 1 | 0 | 1 | 1 | 1 | 1 | unclear | 8 |
| Youssef N (48) | 1 | 1 | 1 | unclear | 1 | 0 | 0 | 0 | 0 | 0 | unclear | 4 |
| Yost MG (20) | 1 | 1 | 1 | unclear | 1 | 0 | 1 | 0 | 0 | 1 | unclear | 6 |
| Yitayih Y (67) | 1 | 1 | 1 | unclear | 1 | 0 | 0 | 1 | 0 | 1 | unclear | 6 |
| Yin Q (49) | 1 | 1 | 1 | unclear | 1 | 0 | 0 | 1 | 0 | 0 | unclear | 5 |
| Weaver MD (4) | 1 | 0 | 1 | unclear | 0 | 0 | 1 | 0 | 1 | 1 | unclear | 5 |
| Wang J (28) | 1 | 0 | 1 | unclear | 1 | 0 | 1 | 1 | 1 | 1 | unclear | 7 |
| Furihata R (51) | 1 | 1 | 1 | unclear | 1 | 1 | 1 | 1 | 1 | 1 | unclear | 9 |
| Rutledge T(33) | 1 | 1 | 1 | unclear | 1 | 0 | 0 | 0 | 1 | 1 | unclear | 6 |
| Chen L (68) | 1 | 1 | 0 | unclear | 1 | 1 | 1 | 0 | 0 | 0 | unclear | 5 |
| Kandemir D (69) | 1 | 1 | 1 | unclear | unclear | 1 | 0 | 1 | 0 | 0 | unclear | 5 |
| Cheng FF(52) | 1 | 1 | 1 | unclear | 1 | 1 | 0 | 0 | 0 | 0 | unclear | 5 |
| Aydin Guclu O (70) | 1 | 1 | 1 | unclear | 1 | 1 | 1 | 0 | 1 | 1 | unclear | 8 |
| Chang Q (71) | 1 | 1 | 1 | 1 | 1 | 1 | 1 | 1 | 1 | 1 | unclear | 10 |
| Mokros KJ (72) | 1 | 1 | 1 | unclear | unclear | 0 | 0 | 0 | 0 | 0 | unclear | 3 |
| Ruggiero JS (30) | 1 | 1 | 1 | unclear | 1 | 0 | 1 | 0 | 1 | 1 | unclear | 7 |
| Vilchez-Cornejo J(40) | 1 | 1 | 1 | unclear | 0 | 0 | 0 | 1 | 1 | 1 | unclear | 6 |
| Sun W (35) | 1 | 0 | 1 | unclear | unclear | 0 | 1 | 1 | 1 | 1 | unclear | 6 |
| Higgins JT (53) | 1 | 1 | 1 | unclear | 1 | 1 | 1 | 1 | 1 | 1 | unclear | 9 |
| Abdelghani M (73) | 1 | 1 | 1 | unclear | 1 | 1 | 0 | 1 | 0 | 0 | unclear | 6 |
| Cai S (41) | 1 | 0 | 1 | unclear | 1 | 1 | 1 | 1 | 1 | 1 | unclear | 8 |
| Abu-Elenin MM (74) | 1 | 1 | 1 | unclear | 1 | 0 | 0 | 0 | 1 | 1 | unclear | 6 |
| Stucky ER (34) | 1 | 1 | 1 | Unclear | 1 | 1 | 0 | 1 | 0 | 0 | Unclear | 6 |
| Lu JL (31) | 1 | 1 | 0 | unclear | unclear | 1 | 0 | 0 | 0 | 0 | unclear | 3 |
| Pang Y (16) | 1 | 1 | 1 | unclear | 1 | 0 | 1 | 0 | 1 | 1 | unclear | 7 |
| Qiao Z(38) | 1 | 1 | 0 | unclear | 1 | 1 | 1 | 0 | 1 | 1 | unclear | 7 |
| Ng A (54) | 1 | 1 | 1 | unclear | 1 | 1 | 1 | 0 | 1 | 1 | unclear | 8 |
| Abbas A (75) | 1 | 1 | 1 | unclear | 1 | 1 | 1 | 0 | 1 | 1 | unclear | 8 |
| Olagunju AT (76) | 1 | 1 | 1 | unclear | 1 | 0 | 0 | 0 | 0 | 0 | unclear | 4 |
| Ding J (55) | 1 | 1 | 1 | unclear | 1 | 1 | 1 | 0 | 0 | 1 | unclear | 7 |
| Hsieh HF (77) | 1 | 1 | 1 | unclear | 1 | 1 | 1 | 0 | 1 | 1 | unclear | 8 |
| Chin W (37) | 1 | 1 | 0 | unclear | 1 | 1 | 1 | 0 | 1 | 1 | unclear | 6 |
| Ibrahim AY (44) | 1 | 1 | 0 | unclear | 1 | 1 | 1 | 1 | 1 | 1 | unclear | 8 |
| Eva Mª Díaz-Ramiro (56) | 1 | 1 | 1 | unclear | unclear | 1 | 0 | 0 | 0 | 0 | unclear | 4 |
| Jiang Y (78) | 1 | 1 | 1 | unclear | 1 | 1 | 1 | 0 | 1 | 1 | unclear | 8 |
| Tu ZH (57) | 1 | 1 | 1 | unclear | 1 | 1 | 0 | 0 | 0 | 1 | unclear | 6 |
| Magnavita N (58) | 1 | 1 | 1 | Unclear | 1 | 1 | 1 | 1 | 1 | 1 | Unclear | 9 |
| Korkmaz S (590) | 1 | 1 | 0 | Unclear | 1 | 1 | 0 | 0 | 0 | 0 | unclear | 4 |
| Dai C (45) | 1 | 1 | 1 | Unclear | 1 | 1 | 1 | 1 | 1 | 1 | Unclear | 9 |
| Geng S (79) | 1 | 1 | 1 | Unclear | 1 | 1 | 1 | 0 | 1 | 1 | Unclear | 8 |
| Ghasemi F (46) | 1 | 1 | 0 | Unclear | Unclear | 0 | 1 | 0 | 1 | 1 | Unclear | 5 |
| Secosan I (60) | 1 | 1 | 1 | Unclear | 1 | 1 | 1 | 0 | 1 | 1 | Unclear | 8 |
| Wang H (63) | 1 | 1 | 1 | Unclear | 1 | 1 | 1 | 1 | 1 | 1 | Unclear | 9 |
| Teo W (61) | 1 | 1 | 1 | Unclear | 1 | 1 | 1 | 1 | 1 | 1 | Unclear | 9 |
| Garcia O (80) | 1 | 1 | 1 | Unclear | 1 | 1 | 0 | 0 | 1 | 0 | Unclear | 6 |
| Koyama F (39) | 1 | 1 | 1 | Unclear | 1 | 1 | 1 | 0 | 1 | 1 | unclear | 8 |
| Aldrees TM(36) | 1 | 1 | 1 | Unclear | Unclear | 1 | 1 | 0 | 1 | 1 | Unclear | 7 |
| Mousavi M (64) | 1 | 1 | 1 | Unclear | 1 | 1 | 0 | 0 | 0 | 0 | Unclear | 5 |
| Peterson U (32) | 1 | 1 | 1 | Unclear | 1 | 1 | 1 | 0 | 1 | 1 | Unclear | 8 |
| Simonetti V (65) | 1 | 1 | 1 | Unclear | Unclear | 0 | 1 | 1 | 1 | 1 | Unclear | 7 |
| Vidott V (42) | 1 | 1 | 1 | unclear | 0 | 1 | 1 | 1 | 1 | 1 | unclear | 8 |
| Tasdemir Yigitoglu G(62) | 1 | 1 | 1 | Unclear | 1 | 1 | 1 | 0 | 0 | 0 | unclear | 6 |
| Ji L (50) | 1 | 1 | 1 | Unclear | 1 | 1 | 1 | 0 | 1 | 1 | Unclear | 8 |
| Aylin Sayilan (47) | 1 | 1 | 1 | Unclear | 1 | 1 | 1 | 1 | 1 | 1 | Unclear | 9 |
|  | | | | | | | | | | | | |

*AHQR questionnaire items:1: Is the source of the data clarified(from investigation or literature review)? 2: Is the inclusion and exclusion criteria of the exposed group and the non-exposed group (cases and controls) listed or refer to previous publications? 3: Is the time period for identifying patients given? 4: If it is not the source of the population, is the research object continuous? 5: Do the subjective factors of the evaluation conceal other aspects of the research object? 6: Are there any descriptions of evaluations performed to ensure quality (such as testing/re-testing of the main outcome indicators)? 7 Were there any explanation of the reasons for excluding any patients from the analysis? 8: Description of that the way to evaluate and/or measure to control confounding factors; 9: If possible, explain how missing data was handled in the analysis; 10: Summarize the patient’s response rate and the completeness of data collection; 11: If there is follow-up, do this study find out what is expected percentage of patients with incomplete data or follow-up results?

**Table2 The result of Newcastle-Ottawa Scale (NOS) evaluated cohort-study**

| Included studies | Identification of research objects | | | | Comparability between groups | Measurement of results | | | Total score |
| --- | --- | --- | --- | --- | --- | --- | --- | --- | --- |
|  | 1 | 2 | 3 | 4 | 5 | 6 | 7 | 8* |  |
| Hillhouse J (27) | 1 | 1 | 1 | 0 | 1 | 1 | 1 | 1 | 7 |
| Wang J (28) | 1 | 1 | 1 | 0 | 2 | 1 | 0 | 0 | 6 |
| Sørengaard TA (19) | 1 | 1 | 1 | 1 | 1 | 1 | 1 | 1 | 8 |
| Fang Y (29) | 1 | 1 | 1 | 1 | 1 | 1 | 1 | 1 | 8 |

*NOS questionnaire items:1: Representativeness of the exposed group; 2: Selection method of non-exposure group; 3. Methods for determining exposure factors; 4.Identify no outcome indicators to be observed at the start of the study; 5.Comparability between exposed and unexposed groups was considered in the design and statistical analysis; 6. The adequacy of the evaluation of the results; 7. Whether follow-up was long enough after the outcome occurred; 8. Whether the follow-up of the exposed group and the non-exposed group is sufficient.

| **Section and Topic** | **Item #** | **Checklist item** | **Location where item is reported** |
| --- | --- | --- | --- |
| **TITLE** | | |  |
| Title | 1 | Identify the report as a systematic review. | P1 |
| **ABSTRACT** | | |  |
| Abstract | 2 | See the PRISMA 2020 for Abstracts checklist. | P2 |
| **INTRODUCTION** | | |  |
| Rationale | 3 | Describe the rationale for the review in the context of existing knowledge. | P3-4 |
| Objectives | 4 | Provide an explicit statement of the objective(s) or question(s) the review addresses. | P4 |
| **METHODS** | | |  |
| Eligibility criteria | 5 | Specify the inclusion and exclusion criteria for the review and how studies were grouped for the syntheses. | P4-6 |
| Information sources | 6 | Specify all databases, registers, websites, organisations, reference lists and other sources searched or consulted to identify studies. Specify the date when each source was last searched or consulted. | P4-5 |
| Search strategy | 7 | Present the full search strategies for all databases, registers and websites, including any filters and limits used. | Appendix 1 |
| Selection process | 8 | Specify the methods used to decide whether a study met the inclusion criteria of the review, including how many reviewers screened each record and each report retrieved, whether they worked independently, and if applicable, details of automation tools used in the process. | P6-7 |
| Data collection process | 9 | Specify the methods used to collect data from reports, including how many reviewers collected data from each report, whether they worked independently, any processes for obtaining or confirming data from study investigators, and if applicable, details of automation tools used in the process. | P6-7 |
| Data items | 10a | List and define all outcomes for which data were sought. Specify whether all results that were compatible with each outcome domain in each study were sought (e.g. for all measures, time points, analyses), and if not, the methods used to decide which results to collect. | P5-6 |
|  | 10b | List and define all other variables for which data were sought (e.g. participant and intervention characteristics, funding sources). Describe any assumptions made about any missing or unclear information. | P6 |
| Study risk of bias assessment | 11 | Specify the methods used to assess risk of bias in the included studies, including details of the tool(s) used, how many reviewers assessed each study and whether they worked independently, and if applicable, details of automation tools used in the process. | P7-8 |
| Effect measures | 12 | Specify for each outcome the effect measure(s) (e.g. risk ratio, mean difference) used in the synthesis or presentation of results. | P8 |
| Synthesis methods | 13a | Describe the processes used to decide which studies were eligible for each synthesis (e.g. tabulating the study intervention characteristics and comparing against the planned groups for each synthesis (item #5)). | P8 |
|  | 13b | Describe any methods required to prepare the data for presentation or synthesis, such as handling of missing summary statistics, or data conversions. | P8 |
|  | 13c | Describe any methods used to tabulate or visually display results of individual studies and syntheses. | P8 |
|  | 13d | Describe any methods used to synthesize results and provide a rationale for the choice(s). If meta-analysis was performed, describe the model(s), method(s) to identify the presence and extent of statistical heterogeneity, and software package(s) used. | P8 |
|  | 13e | Describe any methods used to explore possible causes of heterogeneity among study results (e.g. subgroup analysis, meta-regression). | P8 |
|  | 13f | Describe any sensitivity analyses conducted to assess robustness of the synthesized results. | P8 |
| Reporting bias assessment | 14 | Describe any methods used to assess risk of bias due to missing results in a synthesis (arising from reporting biases). | P7-8 |
| Certainty assessment | 15 | Describe any methods used to assess certainty (or confidence) in the body of evidence for an outcome. | P7 |
| **RESULTS** | | |  |
| Study selection | 16a | Describe the results of the search and selection process, from the number of records identified in the search to the number of studies included in the review, ideally using a flow diagram. | P8-9 |
|  | 16b | Cite studies that might appear to meet the inclusion criteria, but which were excluded, and explain why they were excluded. | Figure 1 |
| Study characteristics | 17 | Cite each included study and present its characteristics. | Table1 |
| Risk of bias in studies | 18 | Present assessments of risk of bias for each included study. | Appendix2 |
| Results of individual studies | 19 | For all outcomes, present, for each study: (a) summary statistics for each group (where appropriate) and (b) an effect estimate and its precision (e.g. confidence/credible interval), ideally using structured tables or plots. | Table and figure showed |
| Results of syntheses | 20a | For each synthesis, briefly summarise the characteristics and risk of bias among contributing studies. | P9 and tables |
|  | 20b | Present results of all statistical syntheses conducted. If meta-analysis was done, present for each the summary estimate and its precision (e.g. confidence/credible interval) and measures of statistical heterogeneity. If comparing groups, describe the direction of the effect. | Figure 2-3 |
|  | 20c | Present results of all investigations of possible causes of heterogeneity among study results. | P14-15 |
|  | 20d | Present results of all sensitivity analyses conducted to assess the robustness of the synthesized results. | P11 and figure 4 |
| Reporting biases | 21 | Present assessments of risk of bias due to missing results (arising from reporting biases) for each synthesis assessed. | P11 and figure 5 |
| Certainty of evidence | 22 | Present assessments of certainty (or confidence) in the body of evidence for each outcome assessed. | P10 and figure 2-3 |
| **DISCUSSION** | | |  |
| Discussion | 23a | Provide a general interpretation of the results in the context of other evidence. | P11 |
|  | 23b | Discuss any limitations of the evidence included in the review. | P14-15 |
|  | 23c | Discuss any limitations of the review processes used. | P14-15 |
|  | 23d | Discuss implications of the results for practice, policy, and future research. | P16 |
| **OTHER INFORMATION** | | |  |
| Registration and protocol | 24a | Provide registration information for the review, including register name and registration number, or state that the review was not registered. | Title page |
|  | 24b | Indicate where the review protocol can be accessed, or state that a protocol was not prepared. | Title page |
|  | 24c | Describe and explain any amendments to information provided at registration or in the protocol. | NA |
| Support | 25 | Describe sources of financial or non-financial support for the review, and the role of the funders or sponsors in the review. | NA |
| Competing interests | 26 | Declare any competing interests of review authors. | Title page |
| Availability of data, code and other materials | 27 | Report which of the following are publicly available and where they can be found: template data collection forms; data extracted from included studies; data used for all analyses; analytic code; any other materials used in the review. | NO |

*From:*  Page MJ, McKenzie JE, Bossuyt PM, Boutron I, Hoffmann TC, Mulrow CD, et al. The PRISMA 2020 statement: an updated guideline for reporting systematic reviews. BMJ 2021;372:n71. doi: 10.1136/bmj.n71

For more information, visit: <http://www.prisma-statement.org/>
